# Change of the work function of platinum electrodes induced by halide adsorption

**DOI:** 10.3762/bjnano.5.15

**Published:** 2014-02-10

**Authors:** Florian Gossenberger, Tanglaw Roman, Katrin Forster-Tonigold, Axel Groß

**Affiliations:** 1 Institute of Theoretical Chemistry, Ulm University, 89069 Ulm, Germany; 2 Helmholtz Institute Ulm (HIU) for Electrochemical Energy Storage, 89069 Ulm, Germany

**Keywords:** density functional theory, ionicity, polarizability, surface dipole, work function

## Abstract

The properties of a halogen-covered platinum(111) surface have been studied by using density functional theory (DFT), because halides are often present at electrochemical electrode/electrolyte interfaces. We focused in particular on the halogen-induced work function change as a function of the coverage of fluorine, chlorine, bromine and iodine. For electronegative adsorbates, an adsorption-induced increase of the work function is usually expected, yet we find a decrease of the work function for Cl, Br and I, which is most prominent at a coverage of approximately 0.25 ML. This coverage-dependent behavior can be explained by assuming a combination of charge transfer and polarization effects on the adsorbate layer. The results are contrasted to the adsorption of fluorine on calcium, a system in which a decrease in the work function is also observed despite a large charge transfer to the halogen adatom.

## Introduction

In electrochemistry, processes at the interface between an electron conductor, the electrode, and an ion conductor, the electrolyte, are studied [1]. In order to be charge neutral, the electrolyte contains equal amounts of anions and cations. In aqueous electrolytes, protons acting as cations are always present [2] whereas halides are often chosen as anions. The contact of a particular solvent with an electrode surface can lead to a rather complex situation at the electrode surface [3–4]. The characteristics of the solvent significantly affects processes such as adsorption and desorption. Because of the strong interaction of halogen atoms with metal electrodes, the metal electrodes typically become halogen-covered through specific adsorption. These adsorbed anions are not only part of the electrochemical double layer, in general they also change the work function of the electrode, which is directly related to the electrode potential [5]. Furthermore, they also affect the chemical properties of the electrodes [6].

In spite of the importance of the specific adsorption of anions in electrochemistry, atomistic details of the role of anions in surface electrochemistry are still poorly understood [7]. Here, surface science studies focusing on the change of the properties of metal surfaces upon halide adsorption can help to elucidate the role of anionic specific adsorption at electrode/electrolyte interfaces, in particular with respect to the adsorption-induced change of the work function. It is known that the work function is strongly influenced by the adsorption of ions, which can lead to both an increase and a decrease of the work function [8–17]. In a previous study, we have addressed the adsorption of iodine and chlorine on Cu(111) [9] by using periodic density functional theory (DFT) calculations. Whereas chlorine causes the expected increase of the work function upon adsorption of an electronegative adsorbate, iodine leads to a surprising decrease of the work function for coverages up to approximately 0.4 ML. By analyzing the underlying electronic structure, we were able to show that this behavior can be explained through a combination of charge transfer and polarization effects of the adsorbate layer.

We have now extended this previous study by considering the adsorption of fluorine, chlorine, bromine and iodine on Pt(111) in order to check whether the findings for halogen adsorption on Cu(111) are also valid for the technologically important electrode material platinum. It has already been observed experimentally [18–20] as well as theoretically [11,13,17] that the adsorption of chlorine, bromine and iodine on Pt(111) leads to an unexpected decrease of the work function. Based on calculations for several adsorbates on tungsten surfaces, Leung, Kao and Su pointed out that it is possible to relate the electronegativity scale to the direction of the charge transfer but not necessarily to the induced work function change. It has also been shown that the formation of halogen oxides at the surface of a metal oxide leads to a decrease in the work function [21]. The problem of the unexpected decrease of the work function was also tackled by Michaelides et al. [8] for a system of nitrogen adsorbed on a tungsten (100) surface. They showed that the decrease of the work function depends strongly on the length of the chemisorption bond. If the adatom is located close to the surface, it is in the region of the overspill electron density of the metal. This leads to an area of electron depletion far from the surface, and in combination with an electron buildup in the area around the adsorbed ion, to a decrease of the work function.

In this paper we present a detailed study of the halogen-induced change of the work function on Pt(111) as a function of the halogen coverage, which has still been missing. We will show that the observed decrease of the work function upon the adsorption of chlorine, bromine and iodine on Pt(111) at low coverages can be explained by the strong polarization of the adsorbed halogen atoms, as in the case of I/Cu(111) [9]. We contrast these results with findings obtained for fluorine adsorption on calcium, for which an adsorption-induced decrease of the work function is also observed. However, due to the particular geometric conditions in this system, the spillout mechanism [8,22] is operative.

## Methods

For the following calculations, the periodic density functional theory (DFT) program Vienna Ab initio Simulation Package (VASP) was used. The exchange and correlation energy was calculated by using the generalized gradient approximation (GGA) with the PBE functional, developed by Perdew, Burke and Ernzerhof [23]. This functional is widely used, as it has been shown to give reliable results in terms of atomization energy, chemisorption energies [24–25], work function changes [26], and good estimates of bulk properties of metals [27]. Hybrid functionals are not necessarily improvements to PBE; for example they do not yield a satisfactory description of the characteristics of transition metals [27].

To describe the ionic cores of the atoms, we used the projector augmented wave potentials (PAW) constructed by Kresse and Joubert [28]. The electronic wave functions were expanded in a plane wave basis set up to an energy cutoff of 400 eV. For the calculations, a periodic slab with a thickness of 7 atomic layers and 4 × 4 lateral periodicity was chosen. All calculations were done by using a symmetric setup of the slab, i.e., the halogen atoms were adsorbed on both sides of the slab, the middle three layers of the slab were kept fixed and the outermost two layers of both sides of the slab together with the adatoms were relaxed. Thus no dipole correction was necessary in order to derive the work function of the surface terminations. The unit cell was computed with a gamma-centered 4 × 4 × 1 *k*-point mesh.

**Figure 1:**
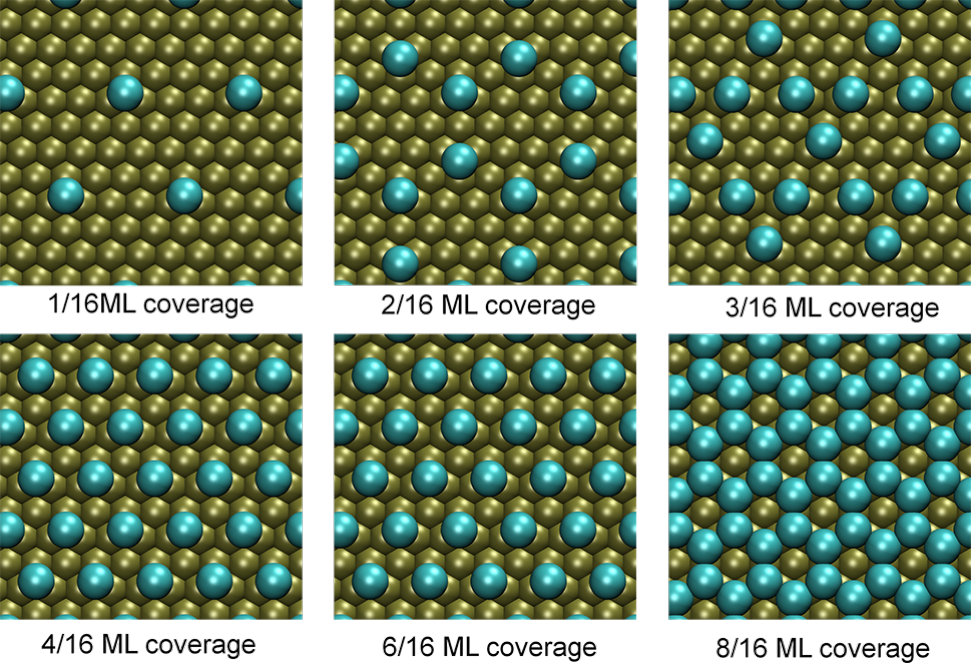
The figures show the relaxed structures of different coverages of chlorine on a Pt(111) surface.

The optimized lattice constant for platinum was found to be *a* = 3.98 Å, which is only 1.48% larger than the standard experimental value [29]. For low coverages the halogens iodine, bromine and chlorine adsorb most stably at the fcc threefold-hollowsite position on a platinum (111) surface. Since the hcp threefold-hollow position is also quite stable, the halogens were ordered in symmetric patterns on the surface with the highest possible nearest neighbor distance to other adsorbed atoms in hcp and fcc positions. The threefold-hollow adsorption positions are considered as the most probable adsorption sites for halogens on metals [9–10,14,30]. In this manner, six different coverages – 1/16 ML, 2/16 ML, 3/16 ML, 4/16 ML, 6/16 ML and 8/16 ML – were created, which are illustrated in Figure 1. The structures of iodine, bromine and chlorine were relaxed completely.

Interestingly enough, fluorine atoms adsorb more stably at the on-top position of platinum. At this position, the average distance to the topmost surface layer is larger than on the threefold-hollow sites. Since we are interested in getting trends among the halogen atoms in order to understand and predict adsorption processes, we kept the fluorine in the threefold-hollow site positions, but allowed for vertical relaxation, which made a better comparison with the results for the chlorine, bromine and iodine adsorption structures possible.

## Results and Discussion

Of central importance for this particular work is the determination of the change of the work function as a function of the halogen coverage. In periodic slab calculations, the work function is given by the difference between the Fermi energy and the value of the one-electron potential in vacuum. Vacuum is reached when the potential does not change anymore with increasing distance from the surface.

**Figure 2:**
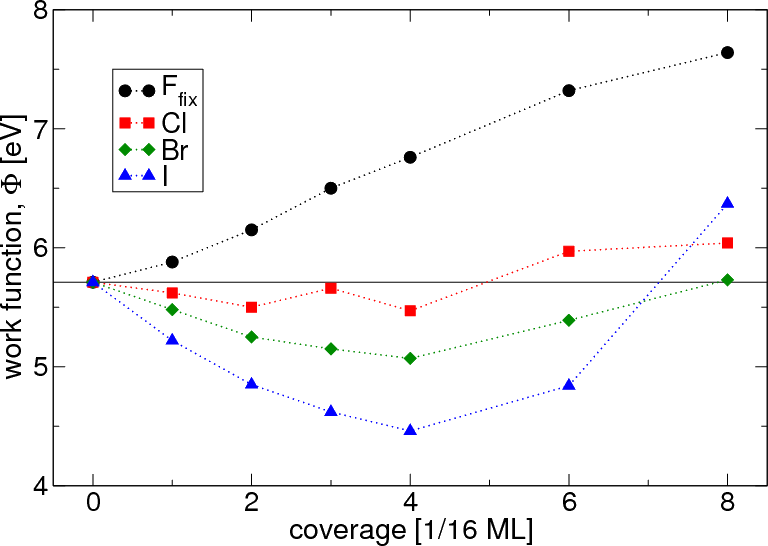
Calculated change of the work function vs coverage for the adsorption of fluorine, chlorine, bromine and iodine on Pt(111). The high value for the 0.5 ML calculation of iodine is due to a double layer structure of the adsorbates, caused by the larger size of iodine atoms.

Figure 2 shows the work function of halogen-covered Pt(111) as a function of the halogen coverage. For clean Pt(111), the calculations yield a value of 5.71 eV. Various experimental measurements in the last decades do not agree well with each other. They are in the range of 5.6 eV to 6.1 eV [20,31–37]. The presence of fluorine on Pt(111) always increases the work function, which is qualitatively consistent with what one expects from a dipole involving a negative charge on the adsorbate. The adsorption of chlorine, bromine or iodine on a platinum (111) surface reduces the work function at low coverages. While the trend reverses at 0.25 ML, ΔΦ becomes positive not until the coverage reaches half a monolayer. The experimental trends [18–20] as well as theoretical values by Migani et al. [10] agree with the calculated results.

Aside from the sign of the work function change, the dependence of ΔΦ on the halogen coverage is another aspect that needs to be clarified. In a simple model, one may completely neglect the interaction between the adsorbates. In this case, a linear trend 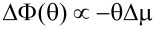 would be expected, where θ is the surface coverage and Δμ is the change in the surface dipole moment brought about by the adsorption of a halogen atom. Obviously, this model is applicable only at low coverages in Figure 2. In a more advanced model, the electrostatic interaction between adjacent dipoles is taken into account by assuming that the mutual repulsion of the dipoles leads to a decrease in the polarity of the halogen–metal bond. The term Δμ thus becomes dependent on the halogen coverage, which causes a saturation of ΔΦ at high coverages. However, this does not explain the observed non-monotonic behavior of the work function change and so a more comprehensive explanation is needed.

In general, an adsorbate layer that involves charge transfer in the adsorption reaction can produce an observable change in the work function of the metal surface since electrons, in leaving the metal surface, will have to pass through the resulting interface dipole layer. Depending on the orientation of the dipole, this can either make removing electrons easier or harder. More precisely, the connection between work function change and surface dipole moment change is given by

**Figure 3:**
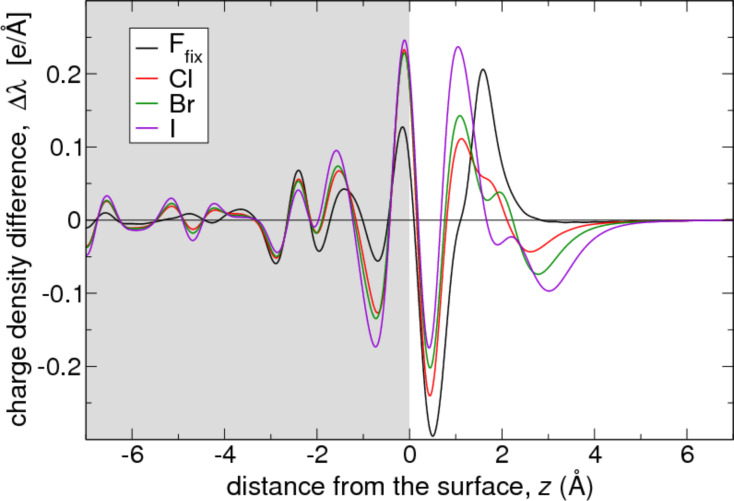
Charge density difference Δλ(*z*) for the adsorption of fluorine, chlorine, bromine, and iodine on Pt(111) at the fcc hollow position for a coverage of 1/16 ML. The subsurface region corresponds to the gray-shaded area at *z* < 0.

[1]
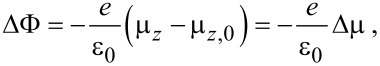


where μ*_z_*_,0_ is the surface-normal dipole moment per unit area of the clean surface, μ*_z_* is the surface-normal dipole moment per unit area for the adsorbate-covered surface. A positive value of μ has traditionally been assigned to a dipole pointing away from the bulk, which leads to a decrease of the work function (ΔΦ < 0). Conversely, a negative μ points into the bulk and increases the work function (ΔΦ > 0). The surface dipole moment changes when the electron density close to the surface becomes redistributed upon bond formation. The most straightforward description of this redistribution is through the electron density difference that is given by the difference of the electron density of the interacting system with the total electron density of the non-interacting metal slab and halogen layer at the same atomic positions, *ρ*_diff_ = *ρ*_Hal+Pt_ − (*ρ*_Hal_ + *ρ*_Pt_). The electron density difference profile Δλ(*z*) along the *z* direction corresponds to the lateral sum of the electron density difference in the *x* and *y* directions,

[2]
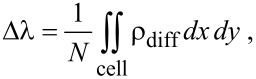


where *N* is the number of halogen atoms adsorbed on one side of the slab per unit cell. The Δλ profiles for a coverage of 1/16 ML of the four halogens are shown in Figure 3. The shape of the diagrams for higher coverages look similar. The profiles illustrate how the electron density is reorganized along the *z* direction when the adatoms adsorb. The gray area on the left hand side denotes the metal slab. The topmost metal atoms are centered at *z* = 0 Å. The electron density difference profile shows a significant electron depletion far from the surface for the case of chlorine, bromine and iodine, followed by an electron buildup close to the surface, and oscillations in the metal. In the case of fluorine, there is just an electron buildup around the fluorine atom, followed by oscillations into the bulk. This electron buildup around the fluorine atom indicates an ionic state. Fluorine is partially constrained to remain at the threefold-hollow sites, where the average distance from the center of the adsorbates to the topmost surface layer is smaller than for fluorine adsorbed at the on-top position. Calculations for F atoms at the most stable adsorption site may give slightly different results in charge transfer and dipole moments.

**Figure 4:**
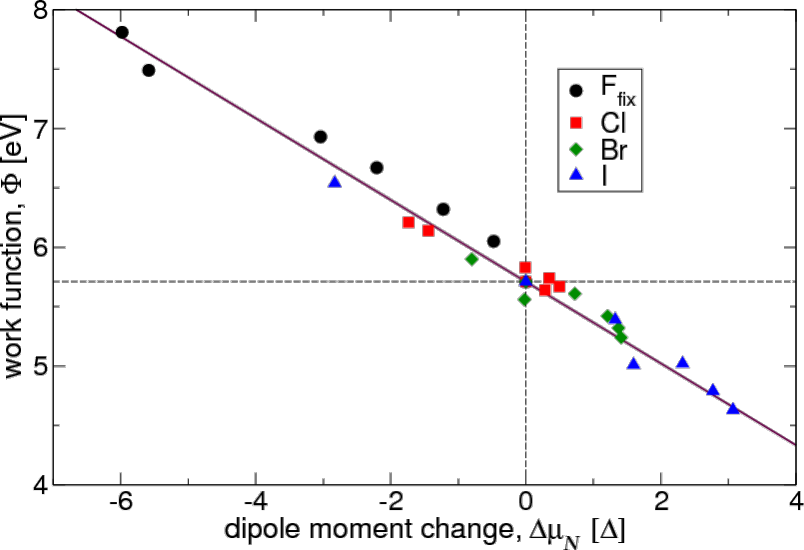
Calculated work function versus dipole moment. The solid line corresponds to the expectation according to Equation 1.

In the next step, the resulting surface dipole moment change Δμ*_N_* can be determined by analyzing Δλ, as in [9] for the adsorption of iodine and chlorine on Cu(111). The *N* indicates that this is the total surface dipole moment of *N* atoms adsorbed in the unit cell. The dipole moment change due to the adsorption process can be calculated by integration of Δλ*_N_*(*z*) = *N*Δλ(*z*) along the *z* direction, perpendicular to the surface,

[3]
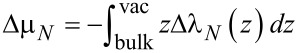


where the negative sign is introduced because positive regions of Δλ*_N_* (i.e., electron buildup) are in fact negatively charged. The integration runs from the central layer of the platinum slab to the middle of the vacuum. Figure 4 shows the good correlation between the calculated work function and the dipole moment derived from the charge distribution, which verifies the assumptions that underlie Equation 3.

Since Δλ of the fluorine-covered platinum slab shows for all coverage values the structure of an electron buildup far from the surface, followed by an electron depletion close to the surface, the dipole moment on each face of the slab becomes more negative as a function of the coverage, which is consistent with a work function increase. For the other three halogens, the electron density difference profile looks more complicated. There is an electron depletion far from the surface, followed by an electron buildup. This structure is sufficiently strong to invert the dipole moment, so that Δμ*_N_* changes sign as a function of the coverage.

It has been suggested that adsorbates that are located rather close to a surface can decrease the electron spillout at the surface. This can cause unexpected changes of the work function, such as the decrease of the work function observed for N on the W(100) surface [8] or the small dipole moment for O on Al(111) [22]. However, the area of electron depletion for chlorine, bromine and iodine is approximately 2.5–4.0 Å away from the center of the topmost platinum atoms, far beyond the region of a sizable electron spillout for the uncovered surface. This electron density shift rather corresponds to a redistribution of the electron density in the adatom layer, which can be associated with a covalent character of the chemisorption bond. This rearrangement is particularly strong for the adsorption of iodine, and slightly weaker for bromine and chlorine. The character of the chemisorption bond between iodine and platinum was discussed in the past [10,17] and conflicting results in terms of the charge of the adatom were presented. In this study, we find that the charge buildup between the iodine and the Pt surface indicates the presence of a covalent bond. Similar conclusions have been found, for example for the adsorption of I on Cu [9] or Cl on Au [14]. Furthermore, in a chronocoulometric study [38] it was found that the adsorbed species is basically a neutral chlorine atom which agrees nicely with our findings. Fluorine, on the other hand, tends to adsorb to the Pt(111) surface mainly in the ionic form.

### Coverage trends

Our calculations confirm the experimental observations [18–20] of a work function minimum as a function of the halogen coverage. Several mechanisms have been proposed to explain its occurrence. For cationic adsorbates, the subsequent increase of Φ beyond the work function minimum was attributed to a reduction of the ionicity of the cationic adsorbate [39]. This explanation, however, does not apply to the halogen adsorption considered here as we still find no indication of cationic adsorption.

The work function minimum has also been explained through differences in site occupancies that occur as halogen coverage increases. Subsurface penetration followed by surface adsorption was one of the possibilities considered in explaining the work function minimum for chlorine on platinum [18]. This was based on the assumption that subsurface penetration and surface adsorption lead to opposite dipole moments at the surface. In contrast, for iodine on platinum, an adsorption site effect was suggested under the assumption that threefold-site adsorption decreases the work function, while adding iodine to top sites increases it [20]. As the coverage increases, more top sites get occupied by iodine, which leads to the increase in Φ beyond the minimum. Still, the change of the surface work function remained negative over the entire coverage range that was considered.

A more recent computational study has shown that the adsorption of isolated iodine atoms at the hollow or top sites both lead to ΔΦ < 0, although the decrease in the work function is larger for the adsorption of iodine at the hollow site [17]. Another explanation for the minimum of the work function was proposed, namely changes in the polarization of the metal substrate. The authors found that the polarization in the platinum substrate that is induced by the presence of the iodine anion adsorbate is reduced with increasing coverage, hence explaining the non-monotonic behavior in ΔΦ.

While changing site occupancy with increasing coverage can and will lead to observable changes in the work function, in this study we focus on changes of the work function that are caused by effects that are primarily electronic in nature, i.e., that are not due to changes in the adsorption or absorption site. Hence a deeper analysis of charge transfer, internal redistribution of charge in the metal substrate, and redistribution of charge on the halogen adatoms is needed. To analyze the surface dipole moments in detail, we use the total surface dipole moment per unit cell normalized to the number of adatoms to define the dipole moment change created per adsorbed atom,

[4]
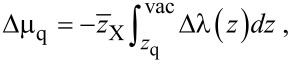


**Figure 5:**
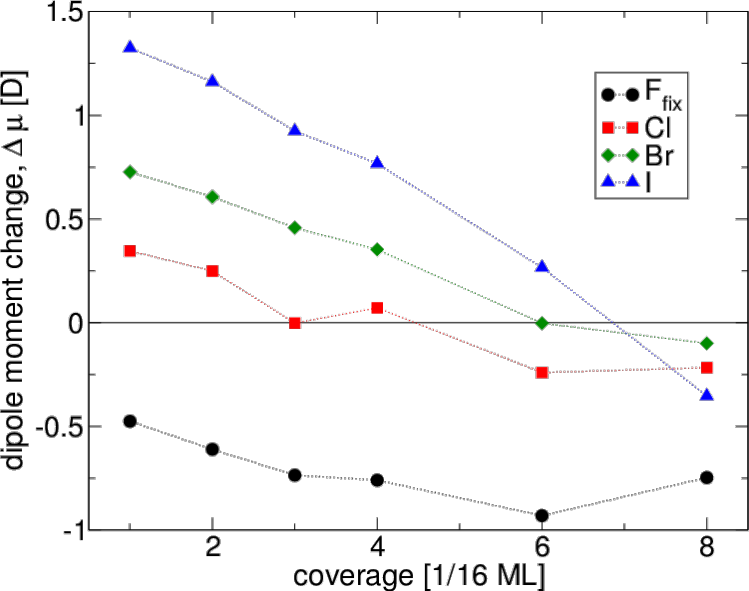
Calculated normalized dipole moment as a function of the coverage of fluorine, chlorine, bromine and iodine on Pt(111).

The normalized dipole moments are shown in Figure 5. The plots are nowhere flat, suggesting the presence of considerable neighboring adatom interactions even at the lowest coverages. There is also a clear tendency for the dipole moment induced by the adsorption of a single halogen atom to be reduced as the concentration of adatoms increases at the Pt surface. Note that the 0.5 ML coverage of iodine is so closely packed that the repulsion of the electron shells induces a two-layer structure of the adsorbate layer. Every second iodine atom becomes a part of a second adsorbate layer, which is positioned about 1.7 Å farther from the surface than the first layer of iodine atoms.

Looking at the charge transfer as a function of the coverage is useful to understand the negative slopes of Δμ for the adsorption of halogens. Quantifying charge transfer between atoms however always involves a more or less ambivalent choice as far as associating the electron density to a particular atom is concerned. We have therefore considered two limits: a maximum-charge-transfer picture, and a zero-charge-transfer picture of halogen adsorption on platinum.

The maximum charge transfer is obtained by assuming that the complete electron buildup between an adatom and the surface is always counted to the adsorbate. In practice, this is done by determining the plane *z* = *z_q_* between the metal and the adatom that maximizes the area under Δλ(*z*) at the halogen side. The charge transfer from the metal surface to the adatoms gives rise to a change of the dipole moment, Δμ_q_. By using a simple model that assumes charge transfer from the topmost Pt layer to the halogen adlayer, the contribution of the electron transfer to the surface dipole moment can be quantified,

[5]
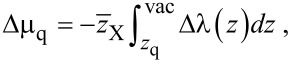


where 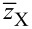 is the average distance of the halogen adatoms from the metal surface. We combine all other parts contributing to the total dipole moment in the term Δμ_pol_, because it involves polarization effects in the metal and in the adlayer. The combination of both contributions leads to the total dipole moment change,

[6]
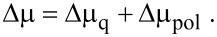


**Figure 6:**
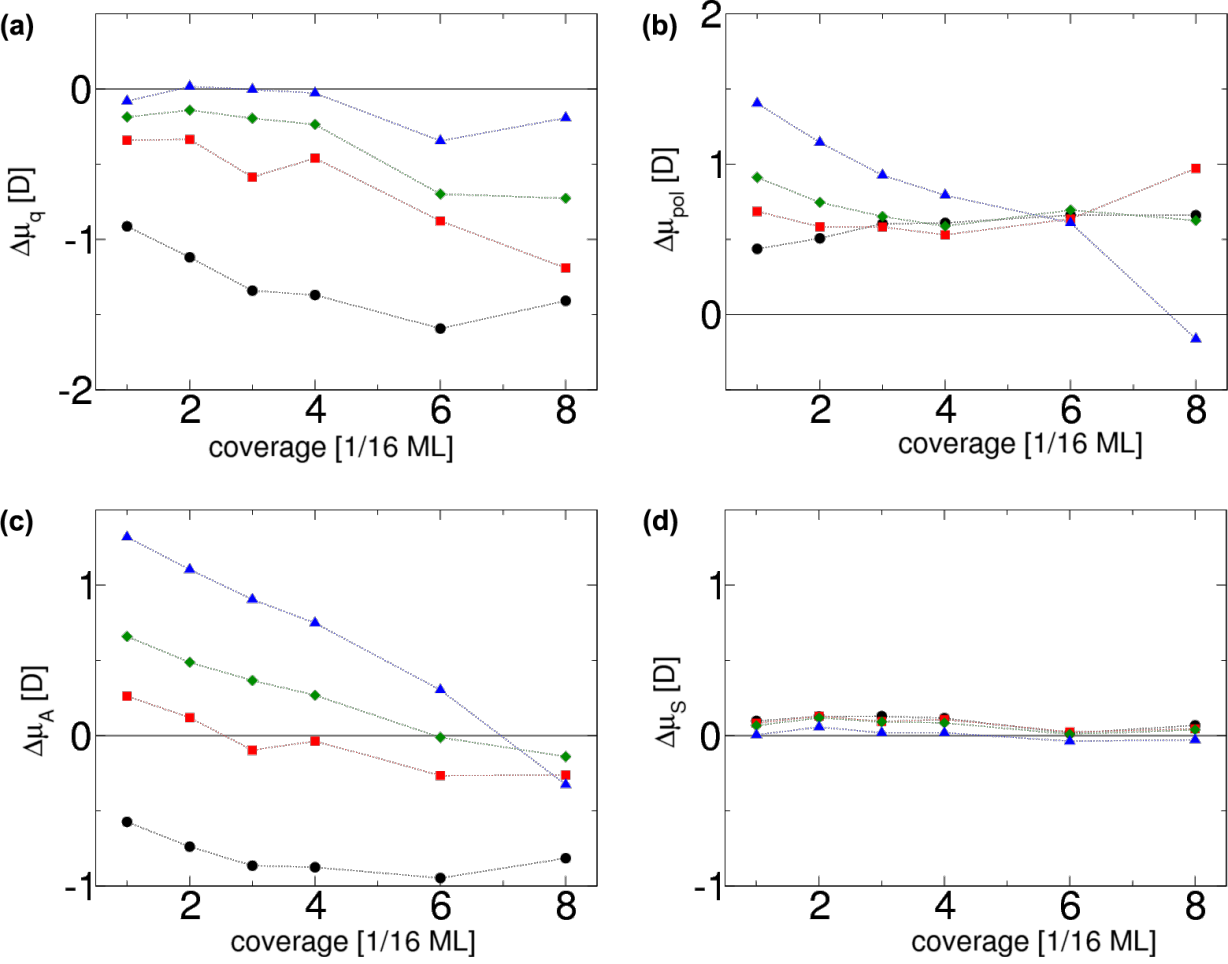
Contributions to the total dipole moment change Δ*μ* according to Equation 6 and Equation 7 as a function of halogen coverage. The term Δ*μ*_q_ describes the purely charge transfer induced dipole moment and Δ*μ*_pol_ the polarization induced dipole moment; Δ*μ_A_* shows the effect of the adsorbate layer on the total dipole moment and Δ*μ*_S_ indicates substrate effects. The color code denoting the different halogen atoms is the same as used in the previous figures.

These contributions are plotted in Figure 6a and Figure 6b, respectively. The effect of the charge transfer Δμ_q_ to the surface dipole is nearly zero for iodine. For fluorine, however, charge transfer plays a significant role, which can be expected since it is more electronegative than the other halogens, as also reported by Migani et al. [10]. Moreover, the negative dipole moment change for the adsorption of fluorine decreases even more with increasing coverage, which is due to the fact that the adsorption distance and charge transfer to the F adatoms increase with increasing coverage.

The results also suggest that higher surface concentrations of adatoms decrease the dipole moment change per adatom through mutual depolarization. This effect is most pronounced for iodine, as well as for a low-coverage adsorption of bromine and chlorine, but not for fluorine because of the low polarizability of small atoms. Besides the repulsion of the dipoles, the electron shells of adsorbed atoms at a higher coverage start to repel.

Another interesting question concerns the importance of the electron density oscillations in the subsurface, as shown in Figure 3. It might be speculated that these oscillations could be responsible for the significant polarization part, Δμ_pol_, of the total dipole moment Δμ. To answer this question, we have divided Δλ into two parts, one representing the dipole moment change due to polarization in the adsorbate layer and the other part representing the dipole moment change due to polarization in the substrate,

[7]
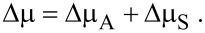


This zero-charge transfer picture for breaking down polarization is especially effective for iodine adsorption on platinum. Such a distinction between pure substrate and adsorbate contributions is again an arbitrary choice. In order to obtain trends, the integration was started from the point *z*_0_, at which the unit cell is divided exactly into the charge neutral part of the adlayer and the charge neutral part of the platinum slab, defined by the condition

[8]
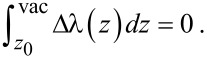


For this choice, the analogous integral on the metal side is also zero due to the overall charge neutrality of the supercell. It is then possible to estimate the surface dipole moment μ_S_ and the adsorbate dipole moment μ_A_ by using

[9]
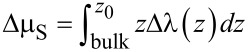


and

[10]
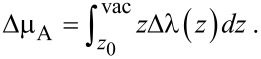


We briefly summarize the difference in the integration limits *z*_q_ and *z*_0_ of Equation 5 and Equation 9, respectively: These equations have the purpose of dividing the unit cell into two parts, but it is not clear where exactly the adatom ends and where the platinum begins or vice versa. The two integration limits mark special points in the graph of Δλ. The limit *z*_q_ divides the unit cell at the point of maximum charge at the adatom, in contrast to *z*_0_ which divides at the point of zero charge at the adatom.

The adsorbate and the substrate dipole moments, which are plotted in Figure 6c and Figure 6d, respectively, indicate that the contribution of the change of the metal substrate dipole moment Δμ*_S_* to the total change of the dipole moment Δμ is minor compared with the impact of adsorbate polarization Δμ_A_, which affects the total dipole moment change quite dramatically. This also means that our analysis does not support the view [17] that substrate polarization plays an important role in explaining the halogen-induced work function decrease.

Additionally, it is noticeable that the decrease in the total change of the dipole moment in the case of iodine and chlorine at around 0.25 ML is much more significant on platinum compared with the total change of the dipole moment of copper [9]. The work function of the copper surface is about 1 eV smaller, thus the charge transfer is larger from copper than from the platinum surface to the halogen adatom. It is for this reason that the adsorption of chlorine on copper does not exhibit a work function minimum with increasing adsorption coverage [9,40], similar to the work function plot of F on Pt in the current study.

### Fluorine on calcium

We have shown that the strong polarizability of large atoms such as iodine leads to a considerable buildup of charge in the adatom–surface bonding regions, which is consistent with covalent bonding, and an accompanying electron depletion region far from the surface which creates a net dipole on the adatom that in turn promotes a decrease in the work function. Here we show that the adsorption of fluorine can also decrease the work function of a metal surface, namely calcium, but through a different mechanism. Calcium is considered to be an attractive electrode material in electrochemical energy storage because of its low electronegativity, earth abundance, and low cost [41]. Fluorine adsorbs stably at a threefold hollow site on calcium, which is a metal with fcc structure and a calculated lattice constant that is 39% larger than that of platinum. At its equilibrium adsorption position, fluorine is only 0.73 Å from the topmost layer of Ca atoms. In contrast, iodine adsorbs 2.07 Å from the platinum surface.

**Figure 7:**
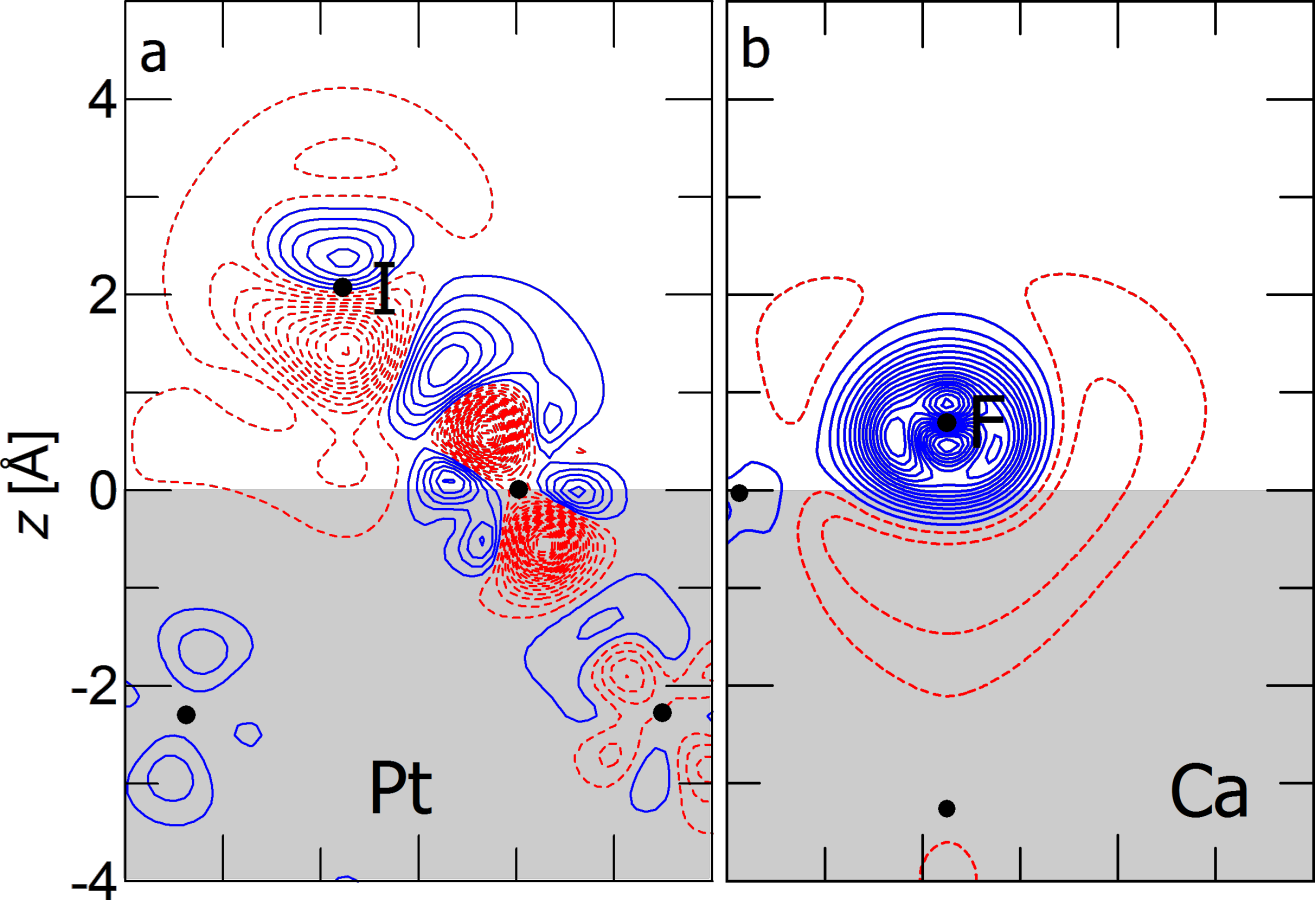
Cross sections of electron density difference ρ_diff_(**r**) at the surface. Solid-blue (dashed-red) contours denote regions of electron buildup (depletion). The interval between contours of constant electron density is 0.01 electrons/Å^3^. The region of the metal slab is shaded gray as a visual aid.

In Figure 7, we compare two systems, in which halogen adsorption decreases the work function of the metal substrate. The left panel shows the adsorption of iodine on Pt(111) at a coverage of 1/9 ML. The right panel shows fluorine adsorption on Ca(111) at a coverage of 1/4 ML. This yields similar absolute values for the coverage per area for the two systems given the stark difference between the lattice constants of Pt and Ca. At these adsorption coverages, iodine reduces the platinum work function by 0.79 eV, while fluorine reduces the calcium work function by 0.20 eV.

Figure 7 shows that halogen adsorption can create a surface dipole that reduces the work function in two very distinct mechanisms, namely adatom polarization and spillout depletion. Iodine on platinum is characterized by a negligible charge transfer, covalent bonding, and polarization on the adatom. There is no evidence for a dominantly ionic bond for I/Pt(111) reported in [17]. Fluorine adsorption on calcium on the other hand is characterized by a large charge transfer to the adatom with negligible polarization, creating a system, which is comprised of a negative ion enveloped by electron depletion. Since fluorine is adsorbed very close to the surface, it is embedded within the electron spillout region of calcium. The depletion of electron density in the spillout region not only reduces the effect of the strongly negative fluorine on the net dipole, but even overcompensates it, resulting in a decrease of the work function.

## Conclusion

The change of the work function induced by halogen adsorption on Pt(111) as a function of the coverage was studied by electronic structure calculations. In general, because of their electronegativity, the adsorption of halogens is associated with a charge transfer from the metal substrate to the adsorbate layer. In the case of fluorine adsorption, this leads to the expected increase in the work function. However, for chlorine, bromine and iodine adsorption on Pt(111), the charge transfer effect is overcompensated by a significant polarization of the adsorbate, causing a work function decrease. The decreasing change of the dipole moment per adatom with an increasing adsorption coverage leads to a maximum in the total surface dipole moment and a minimum in the work function at a coverage of approximately 0.25 ML. The mutual depolarization within the adsorbate layer contributes to the eventual work function increase.

The anomalous work function change on platinum is large because of the high work function of clean platinum, which favors only a small electron transfer to the halogen adatoms compared with other metals. Therefore, polarization effects that reverse the dipole moment attributed to charge transfer are more pronounced than on metals with smaller work functions such as copper.

Furthermore, we showed that fluorine adsorption can also lead to an anomalous work function decrease, but through a different mechanism. On calcium, fluorine is adsorbed close to the surface because of the large spacing between the calcium atoms. This causes a depletion of the electron density in the spillout region, which results in a decrease of the work function.
